# Depot-Dependent Effects of Adipose Tissue Explants on Co-Cultured Hepatocytes

**DOI:** 10.1371/journal.pone.0020917

**Published:** 2011-06-07

**Authors:** Zhen-Yu Du, Tao Ma, Erik-Jan Lock, Qin Hao, Karsten Kristiansen, Livar Frøyland, Lise Madsen

**Affiliations:** 1 National Institute of Nutrition and Seafood Research (NIFES), Bergen, Norway; 2 Department of Biology, University of Copenhagen, Copenhagen, Denmark; Universidade Federal do Rio de Janeiro, Brazil

## Abstract

We have developed an *in vitro* hepatocyte-adipose tissue explant (ATE) co-culture model enabling examination of the effect of visceral and subcutaneous adipose tissues on primary rat hepatocytes. Initial analyses of inflammatory marker genes were performed in fractionated epididymal or inguinal adipose tissues. Expressions of inflammation related genes (IL-6, TNF-α, COX-2) were higher in the inguinal than the epididymal ATE. Similarly, expressions of marker genes of macrophage and monocyte (MPEG-1, CD68, F4/80, CD64) were higher in the stromal vascular fraction (SVF) isolated from inguinal ATE than that from epididymal ATE. However, expressions of lipolysis related genes (ATGL, HSL, perilipin-1) were higher in the epididymal adipocytes than inguinal adipocytes. Moreover, secretion of IL-6 and PGE_2_ was higher from inguinal ATEs than from epididymal ATEs. There was a trend that the total levels of IL-6, TNF-α and PGE_2_ in the media from inguinal ATEs co-cultured with primary rat hepatocytes were higher than that in the media from epididymal ATEs co-cultured with hepatocytes, although the significant difference was only seen in PGE_2_. Lipolysis, measured as glycerol release, was similar in the ATEs isolated from inguinal and epididymal adipose tissues when cultured alone, but the glycerol release was higher in the ATEs isolated from epididymal than from inguinal adipose tissue when co-cultured with hepatocytes. Compared to epididymal ATEs, the ATEs from inguinal adipose tissue elicited a stronger cytotoxic response and higher level of insulin resistance in the co-cultured hepatocytes. In conclusion, our results reveal depot-dependent effects of ATEs on co-cultured primary hepatocytes, which in part may be related to a more pronounced infiltration of stromal vascular cells (SVCs), particularly macrophages, in inguinal adipose tissue resulting in stronger responses in terms of hepatotoxicity and insulin-resistance.

## Introduction

Obesity is the major factor predisposing individuals to a complex of dyslipidemic/metabolic disorders collectively named the metabolic syndrome. An important early event preceding overt symptoms of the metabolic syndrome is the unnoticed development of insulin resistance. Obesity can be considered as a chronic low-grade inflammatory state characterized by infiltration of macrophages, and high expression of inflammatory markers and adipokines in macrophages and adipocytes [1], [2], [3]. Additionally, elevated levels of circulating free fatty acids (FFA), mainly liberated from adipocytes in obese individuals, are recognized as a significant contributor to insulin resistance pathophysiology [4]. The elevated level of FFA in circulation also leads to accumulation of lipid in non-adipose tissues resulting in cellular dysregulation and functional impairment in liver [5] and muscle [6]. This kind of FFA-induced insulin resistance and cellular toxicity has been referred to as “lipotoxicity” [7].

The individual contributions of different adipose tissue depots in the development of systemic insulin resistance remain to be fully elucidated. Central obesity caused by increased amount of intra-abdominal fat (visceral fat) is commonly considered to be associated with insulin resistance, high risk of type 2 diabetes, dyslipidemia and high mortality [8], [9]. By contrast, increasing the amounts of subcutaneous fat, particularly in gluteofemoral regions, is associated with improved insulin sensitivity and lower risk of development of type 2 diabetes, compared to the levels in the central obesity [10]–[13]. However, FFA released from visceral fat to liver only accounts for 5–10% and 20–25% of total FFA delivery in lean and obese subjects, respectively [14]. Thus, the contribution of visceral lipolysis to systemic FFA availability is suggested to account for less than 5% of the total FFA burden [14], and it has been argued that subcutaneous fat, especially in the upper body is the major contributor to the supply of FFAs to extrahepatic tissues in insulin-resistant states [15]. Moreover, other factors, such as the depth of adipose tissue location and circulation, are also associated with systemic insulin resistance [16].

Co-culture systems serve as useful tools to obtain insights into the cross-talk between different cell types. For instance, adipocyte-myocyte/neuron/hepatocyte co-culture systems have been used successfully in the investigation of the functions of adipokines [17]–[19]. However, in addition to adipocytes, adipose tissue contains a large fraction of other cell types (stromal-vascular cells, SVCs), such as pre-adipocytes, lymphocytes, macrophages, fibroblasts and vascular cells. In keeping with the notions that i) the abundance of macrophages is increased in adipose tissue of obese individuals [20], [21] and ii), pro-inflammatory cytokines such as TNF-α, IL-6 and MCP-1, which are all causally linked to development of insulin resistance, are produced by both adipocytes and infiltrating macrophages [22]–[24], the physiological relevance of pure adipocytes in co-culture systems is limited. Additionally, adipose tissues have different degrees of vascularization, which could enhance the inflammatory properties of the cells near the micro vessels [25]. Using explants of rat epididymal and inguinal white adipose tissues in co-culture with rat primary hepatocytes, we demonstrate depot-dependent effects of adipose tissue explants on the co-cultured hepatocytes in relation to the development of hepatic insulin resistance and cytotoxicity.

## Materials and Methods

### Ethics Statement

This study didn't contain human materials and it was not performed in living animals. The animals used for isolation of hepatocytes and adipose explants were handled in accordance with local institutional recommendations and the entire protocol was reviewed by local ethics board of National Institute of Nutrition and Seafood Research (NIFES).

### Materials and animals

[1-^14^C] oleic acid and [U-^14^C] glucose were obtained from Moravek Biochemicals, Inc. (Mercury Lane Brea, CA, USA). L-carnitine, bovine serum albumin (BSA) and other biochemicals were from Sigma. The DMEM medium used for tissue culture was from Invitrogen Co. (Carlsbad, CA). Healthy male Wistar rats (8 weeks, 250–300 g) were purchased from Taconic (Bomholtvej, Danmark) and kept at 23°C in a light-controlled room (light:dark, 12 h:12 h) with free access to tap water and standard laboratory chow (SDS, Essex, England). Before tissue isolation, the rats were anesthesized with isoflurane and killed by exsanguination.

### Hepatocytes and adipose tissue explants (ATEs) isolation and co-culture

**Figure 1:**
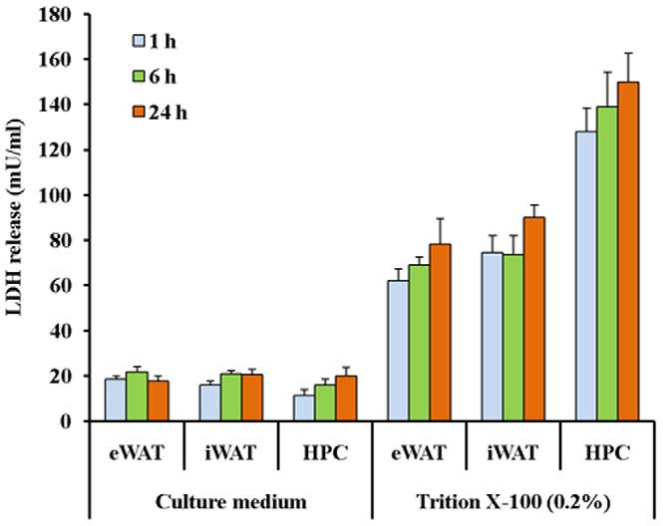
Cellular viability of primary hepatocytes and explants of epididymal adipose tissue (eWAT) and inguinal adipose tissue (iWAT) during 24 h culture. Culture medium containing Trition X-100 (0.2%) was used as a positive control.

HPCs were isolated by collagenase perfusion and purified using Percoll gradients as described [26], [27]. The cells were plated in the DMEM (glucose 4500 mg/L, pyruvate free) supplemented with 10% fetal bovine serum, and 1% (v/v) antibiotic antimycotic solution in 6-well plates with 2 million cells and 4 ml medium per well, and pre-incubated at 37°C for 3 h in a humidified atmosphere of 5% CO_2_–95% air. After pre-incubation, culture media were removed and the HPC monolayers were washed with pre-heated DMEM for 2 times to eliminate unattached cells. DMEM containing 10% BSA and 1% (v/v) antibiotic antimycotic solution was used for further culture. During the pre-incubation of HPCs, eipididymal and inguinal adipose tissues were collected from rats and cut into small pads (10 mg each) above an ice-cooled stainless steel plate. These adipose tissue explants (ATEs) were rinsed three times by DMEM containing 10% BSA and 1% (v/v) antibiotic antimycotic solution, and 20 mg of ATEs were placed in the culture wells and co-cultured with the HPCs. The individual HPCs culture was regarded as the control. The ratio of ATEs to HPCs (10 mg ATEs/million HPCs) was determined in preliminary experiments, which indicated a ratio with more than 50 mg ATEs/million HPCs would induce complete cell death of all co-cultured HPCs within 24 h (data not shown). For individual ATE cultures, the ATEs were placed in DMEM containing 10% BSA in a humidified atmosphere of 5% CO_2_–95% air. Under these conditions ATEs remain viable and functional for at least 48 h [28], [29]. The viability of primary HPCs and ATEs after 24 h of incubation was also documented by the unchanged pH value (data not shown) and activitiy of lactate dehydrogenase (LDH) in the medium after 1, 6, and 24 h. Inclusion of Triton X-100 (0.2%) was used as the positive control (Fig. 1).

### Histological study

The freshly prepared ATEs were immediately put in freshly-made ice-cold 4% paraformaldehyde (0.1 M phosphate buffer) and fixed for 24 hours at 4°C. The samples were dehydrated in graded series of ethanol, followed by xylene (2 times) and embedded in paraffin. Paraffin blocks were sectioned (7µm) and sections deparaffinized, dehydrated and stained with haematoxylin and eosin following a standard procedure. At least 50 sections per tissue were evaluated.

### Assessment of cytotoxic environment in co-culture

The cytotoxic environment in co-culture was evaluated by the number of detached HPCs, activity of LDH in extracellular media, and DNA fragmentation of the attached HPCs. At different time points, the culture dishes were shaken mildly to secure an even distribution of cells in the medium, and then aliquots of media were collected to count the number of detached cells. Afterwards, these media aliquots were centrifuged by 2000 g×5 min, and the supernatants were collected for determination of LDH activity using a Roche reagent kit (Roche Norge, Oslo, Norway). DNA fragmentation of the attached HPCs was assayed by using a DNA ladder isolation kit (EMD Chemials Inc., Darmstadt, Germany).

### Total FA β-oxidation capacity of the HPCs after co-culture

The mitochondrial function of the attached HPCs was determined by assaying oleic acid β-oxidation. After co-culture for 24 h, the HPCs monolayers were washed gently with pre-heated PBS. Total FA β-oxidation capacity of the HPCs was measured as described previously [30]. The reaction was initiated by the addition of 3 ml of DMEM (pH 7.4) containing 0.5 mM L-carnitine and 0.2 mM [1-^14^C] oleic acid (0.45 Ci/mol) as potassium salt, bound to BSA in a 4∶1 molar ratio. After incubation in a humidified atmosphere (5%CO_2_, 95% air) for 1 h at 37°C, 0.5 ml of 1% of Triton X-100 was added followed by 3 ml of 10% (w/v) perchloric acid to stop the reaction. A small plastic tube with a flat bottom containing 0.4 ml of Hyamine (Packard, Meriden, CT) was immediately placed into the culture dish to trap the ^14^CO_2_ released from the acidified medium. After 90 min, media were filtered using Millipore filters (0.45 µm pore size) under very low pressure. Both 0.5 ml from each filtrate containing the acid-soluble products and ^14^CO_2_ trapped into Hyamine was determined using an Ultima Gold XR (Packard).

### Insulin-stimulated glycogen synthesis in the HPCs after co-culture

The analysis of insulin-sensitive glycogen synthesis of the attached HPCs was modified from that described previously [19], [31]. Briefly, after co-culture, the attached HPCs were incubated in 2 ml DMEM containing 5.6 mM [U-^14^C] glucose (final dose 4 µCi/ml), 10 nM dexamethasone and 100 nM insulin at 37°C. After 2 h, the reaction was terminated by washing the cells three times with ice-cooled PBS. The cells were solubilized with 2 ml of 10 N KOH, and 5 mg of cold glycogen carrier was added to the lysates. Total glycogen in the lysates was precipitated with ethanol (final conc. 63%, v/v) overnight and recovered by centrifuged at 3000×g for 15 min. After washing with 63% ethanol once, the glycogen pellets were dissolved in hot water and the radioactivity was determined. The insulin-free reaction was regarded as the basal level.

### Assessment of inflammatory factors in the culture medium

During individual ATEs culture or HPC/ATE co-culture, 200 µl of medium aliquots from each well were collected. Before the measurement, all aliquots of medium were centrifuged by 5000 g ×10 min at 4°C. The supernatant was analyzed by interleukin-6 (IL-6) and tumor necrosis factor-α (TNF-α) ELISA kits (Invitrogen Co., CA), prostaglandin E_2_ (PGE_2_) immunoassay kit and glycerol kit (Cayman Chemical Co.), respectively.

### Isolation of the stromal vascular fraction and adipocytes

The stromal vascular fraction was isolated from ATEs dissected out from mice as earlier described [32], with minor modifications. Freshly collected ATEs from epididymal and inguinal adipose tissues were digested with 5% collagenase type I (Sigma C9722) and 10% FBS dissolved in DMEM for 40 minutes at 37°C under constant shaking. The digested ATEs were centrifuged at 600×g for 5 minutes at room temperature. The fat layer on top was washed by fresh DMEM three times and collected as the total adipocytes fraction. The precipitated white SVCs was washed by DMEM three times and collected as the SVCs fraction. All fractions were immediately used for RNA extraction.

### RNA extraction and gene expression

#### i) From HPCs after co-culture

After co-culture for 24 h, the fat pads and culture media were removed. The HPCs monolayers were washed by pre-heated PBS solution, and then total mRNA was extracted by using Trizol reagent (Gibco-BRL, Gaithersburg, MD) according to the protocol provided by the manufacturer. The quality of the isolated RNA was assessed by the 260/280 nm absorbance ratio. Total mRNA was reverse-transcripted into cDNA and was quantified by real-time quantitative PCR (ABI 7500, Applied Biosystems, Foster City, CA). The expressions of the housekeeping genes, beta-actin and GAPDH, were used for normalization. Primer pairs of target genes were designed by using Primer Premier 5.0 (Premier Biosoft International, Palo Alto, CA) and synthesized by Invitrogen Co. (Carlsbad, CA). The full names of genes and the sequences of all primers are shown in Table 1.

**Table 1:** Primers used for analysis of gene expression.

Genes	5′-sense primer-3′5′-antisense primer-3′
*Inflammatory factor related*	
Interleukin-6 (IL-6)	TTCCAGCCAGTTGCCTTCTT TGTTGTGGGTGGTATCCTCTGT
Tumor necrosis factor-α (TNF-α)	ACGGAAAGCATGATCCGAGAT GCCACGAGCAGGAATGAGAA
Cyclooxygenase-2 (COX-2)	GGAGGAGAAGTGGGGTTTAGGA TGCGTTGATGGTGGCTGTC
*Stromal-vascular fraction mark genes*	
Macrophage expressed gene 1 (MEPG-1)	ATTCAGGGCTTATTGGTGCG GACTGTGCATTTGTCATTGGGT
Cluster of differentiation 68 (CD68)	ATGCCACAGTTTCTCCCACC TGTAGTTTCCAAGAGCCCCAGT
Macrophage migration inhibitory factor (MIF)	CGGACCGGGTCTACATCAACTA GCAGCAAGACTCGAAGAACAGC
F4/80	TGGAATGCATAATCGCTGCT CAAGGAGGGCAGAGTTGATC
Cluster of differentiation 64 (CD64)	CACCGTGAAAGAGCTATTTGC AAGTAAAGCCGTAAGCCAGG
Adipose differentiation related protein (ADRP, or Perilipin-2)	TGGGTGGAGTGGAAGAGAAT ATGTGACTCGATGTGCTCAG
Leucine-rich alpha-2-glycoprotein 1 (LRG1)	CATCAAGGGAGAACCCTGTT CCGACTGCAGTATCAAGCAT
Myeloperoxidase (MPO)	TACGGGATGGCGATAGGTTT TGTTTACTGAACACACCCGG
*Adipose tissue lipolysis related*	
Adipose triglyceride lipase (ATGL)	TGGATGGCGGCATTTCA GTTCGTGGATGTTGGTGGAG
Hormone sensitive lipase (HSL)	AGCGCCTATTCAGGGACAGA CCAGGAAGGAGTTGAGCCAT
Perilipin-1	GTACACTATGTCCCGCTTCC CCACCTCTGCTGGAGGATTA
*Adipokine genes*	
Adiponectin	GGGACAACAATGGACTCTATGC GTTCTTTGATTCTCGGGGCTA
Lipocalin-2	GACAACTGAACAGACGGTGAGC CTGGCAACAGGAAAGATGGA
*Hepatocyte apoptosis related*	
Caspase-3	TGACGACAGGGTGCTACGAT TGAGGCTGCTGCATAATCG
Caspase-8	GAAGAACTGGCTGCCCTCAA GGACAAATTGTCTTCCTCCAAC
B-cell lymphoma 2 (BCL-2)	TGTGGCCTTCTTTGAGTTCG ATCCCAGCCTCCGTTATCCT
*Hepatocyte insulin-sensitive glycogen synthesis related*	
Insulin receptor substrate 1 (IRS-1)	TCTACACCCGAGACGAACAC GGCCTTTGCCCGATTAT
Glucose transporter 2 (GLUT-2)	ACACCAGCACATACGACACCA CAAAGAACGAGGCGACCATT
Glycogen synthase 2 (GYS-2)	ACTCCAAACGGCTTGAACG GCCATAGAAATGACCTCGAACA
*Reference genes*	
β-actin	AGGGAAATCGTGCGTGAC CGCTCATTGCCGATAGTG
Glyceraldehyde 3-phosphate dehydrogenase (GAPDH)	GGCAAGTTCAACGGCACAGT CGCCAGTAGACTCCACGACATA
TATAA-box binding protein (TBP)	CCACCGTGAATCTTGGCTGTA ACGCAGTTGTTCGTGGCTCT

#### ii) From adipose tissues

The whole ATEs, total adipocytes and SVCs fraction were homogenized in Trizol reagent. The extraction of total mRNA, cDNA transcription and real-time qPCR process were as described above. TATAA-box binding protein (TBP) and beta-actin were used as the reference genes. The full names of genes and the sequences of all primers are shown in Table 1.

### Statistical analysis

The data presented are means ± S.D. of three independent experiments. Statistical differences of mean values were tested by one way ANOVA and the significant difference between two given groups was analyzed using Student's *t*-test (SPSS 9.0, SPSS Inc., Chicago, IL, USA).

## Results

### Depot-dependent gene expression in adipose tissues

**Figure 2:**
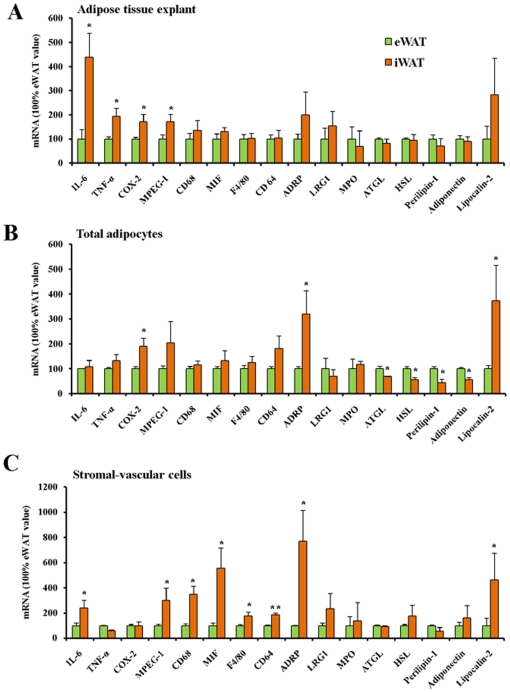
Depot-dependent genes expression in rat epididymal adipose tissue (eWAT) and inguinal adipose tissue (iWAT), using whole adipose tissue explants, total adipocytes and stromal-vascular cells fractions, respectively. TATAA-box binding protein (TBP) and beta-actin were used as reference genes. The abbreviations are explained in Table 1. * and **, *P*<0.05 and *P*<0.01 *vs* eWAT value, respectively.

Pro-inflammatory adipokines/cytokines, eicosanoids and FFA, produced by adipose tissues contribute to the development of insulin resistance in the liver. In order to evaluate the possible different contributions of different adipose tissue depots, the depot-dependent expression of genes related to production of inflammatory factors and lipolysis was firstly measured in the whole ATEs from iWAT and eWAT. As shown in Fig.2A, the expressions of pro-inflammatory factors such as IL-6, TNF-α and cyclooxygenase-2 (COX-2) were significantly higher in iWAT than in eWAT. Expressions of the lipase genes, adipose triglyceride lipase (ATGL), hormone sensitive lipase (HSL) and the lipid droplet scaffold protein, perilipin-1, as well as adiponectin and lipocalin-2 genes were similar in eWAT and iWAT. The expression of the macrophage expressed gene 1 (MPEG-1) was significantly higher in iWAT than eWAT (Fig. 2A), and this was accompanied by a tendency towards higher expressions of the other macrophage markers CD68 and macrophage migration inhibitory factor (MIF) in iWAT than in eWAT. Moreover, leucine-rich alpha-2-glycoprotein 1 (LRG1) and myeloperoxidase (MPO), which are specific neutrophil markers, had non-significant differences between iWAT and eWAT (Fig 2A). In order to investigate the individual contribution of adipocytes and SVCs to gene expression in intact ATEs, expressions of the same genes were also determined in isolated total adipocytes and SVCs, respectively. With exception of COX-2, expressions of most inflammatory markers and neutrophil markers were comparable in total adipocytes isolated from eWAT and iWAT (Fig. 2B). Expressions of periplin-2 (ADRP) and lipocalin-2 were higher in iWAT than in eWAT, whereas significantly higher expressions of ATGL, HSL, perilipin-1 and adiponectin were found in eWAT compared with iWAT. This indicates adipocytes isolated from eWAT have higher lipolytic capacity than the adipocytes in iWAT. As expected, more differences of inflammation related genes between two adipose tissues were observed in SVCs than in adipocytes (Fig 2C). Except for TNF-α and COX-2, almost all inflammatory marker genes were expressed at higher levels in SVCs isolated from iWAT than eWAT (Fig 2C), particularly the genes selectively expressed in macrophages (MPEG-1, CD68, F4/80) and monocyte/neutrophil (CD64). No significant differences were seen in neutrophil marker genes between SVCs of two adipose tissues. The expression of lipocalin-2, an adipokine promoting insulin resistance [33], was far higher in SVCs from iWAT than eWAT. However, the differences of lipase related genes in adipocytes were not found in SVCs. Together, these results imply that the higher expression of inflammatory markers in iWAT than eWAT to a large extent is due to higher levels of SVCs, such as macrophages and monocytes, in iWAT.

### Histological characteristics of eWAT and iWAT

**Figure 3:**
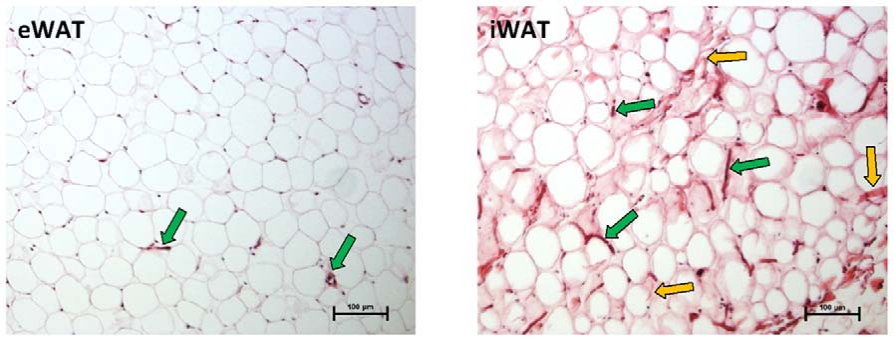
Histological characteristics of rat epididymal adipose tissue (eWAT) and inguinal adipose tissue (iWAT). Standard H&E staining of sections was performed. As compared with eWAT, less adipocytes, bigger intercellular space, more blood cells/vessels residue (deep red particles/tubes with green arrow) and abundant collagen fibers (light red filaments with yellow arrow) were seen in iWAT.

The distinct gene expression patterns of eWAT and iWAT indicate potential functional and morphological differences between the two adipose tissue depots. To demonstrate that a larger fraction of cells in iWAT is comprised of non-adipocytes, sections of both eWAT and iWAT were stained by H&E. As shown in Fig 3, large morphological differences were observed. In eWAT, typical adipocytes were arranged in close contact with each other and only few micro vessels were observed. In iWAT, larger intercellular spaces were present between the adipocytes and compared with eWAT, more micro vessels and abundant collagen fibers were seen. Together, histological examination and gene expression analyses of fractionated adipose tissue suggest that iWAT contains more SVCs, such as macrophages, neutrophils and fibroblasts, which are commonly believed to secret higher levels of pro-inflammatory cytokines and eicosanoids than adipocytes.

### Release of inflammatory factors in individual cultures and co-culture of ATEs and HPCs

**Figure 4:**
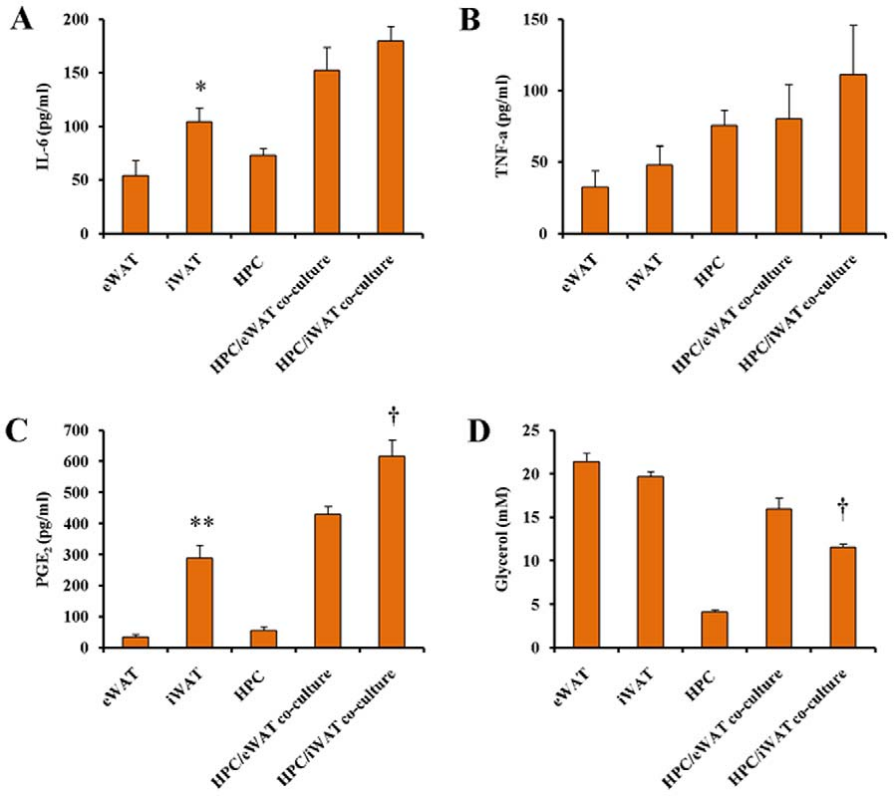
Medium concentration of inflammatory factors in individual and co-culture of rat hepatocytes and adipose tissue explants after 24 h. As described in the Material and Methods section, rat hapatocytes were co-cultured with 20 mg of adipose tissue explants (10 mg ×2 pieces) from rat epididymal adipose tissue (eWAT) or inguinal adipose tissue (iWAT) in 4 ml DMEM medium and cultured at 37°C. The individual culture of explants of eWAT, iWAT and hepatocytes were also performed. After 24 h, the medium concentrations of IL-6 (A), TNF-α (B), PEG_2_ (C) and glycerol (D) were measured. * and **, *P*<0.05 and *P*<0.01 *vs* eWAT group value, respectively. †, *P*<0.05 *vs* eWAT co-culture group value.

To investigate whether higher expression of macrophage/monocyte selective and inflammatory marker genes in iWAT and eWAT translated into different production of pro-inflammatory mediators, we measured the release of IL-6, TNF-α, PGE_2_ and glycerol in individual cultures of the ATEs isolated from iWAT (iATEs) and eWAT (eATEs). As shown in Fig. 4, iATEs secreted significantly more IL-6 and PGE_2_ than eATEs after 24 h of incubation. A tendency towards higher TNF-α release from iATEs culture than eATEs culture was also seen. Glycerol release, an indicator of lipolysis, was similar from eATEs and iATEs, although a tendency of higher level in eATEs than iATEs was noticed.

Co-culture with primary hepatocytes (HPCs) might affect the secretion of inflammatory factors. To examine this, we measured the release of IL-6, TNF-α, PGE_2_ and glycerol into media from HPC alone and HPC/ATE co-cultures. As expected, the concentrations of IL-6, PGE_2_ and glycerol in the media from HPC/ATE co-cultures were significantly higher than those in the media from HPC cultures (Fig 4). Secretion of TNF-α was, however, not significantly higher from HPC/ATE co-cultures than that from HPC cultures. There was a trend that the media from HPC/iATE co-culture contained more IL-6, TNF-α and PGE_2_ than media from HPC/eATEs co-culture, but a significant difference was only observed with respect to PGE_2_ (Fig. 4). On the other hand, the media of HPC/eATE co-culture contained significantly more glycerol than the media of HPC/iATE co-culture. Together, these results suggest that iATEs secrete more inflammatory cytokines and eicosanoids than eATEs, whereas eATEs have higher lipolytic rate and secrete more FFA than iATEs.

### Cytotoxic properties in HPC/ATE co-culture

**Figure 5:**
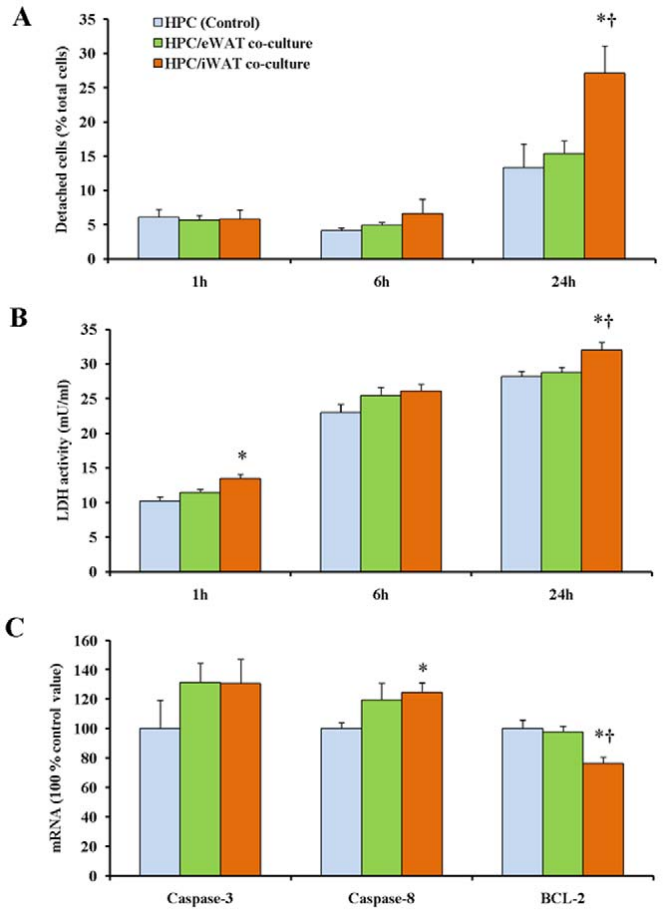
Cytotoxic properties of rat hepatocytes (HPC) co-cultured with adipose tissue explants from epididymal adipose tissue (eWAT) and inguinal adipose tissue (iWAT). A: Detached HPCs in HPC-fat pads co-culture after 24 h. B: Activity of lactate dehydrogenase (LDH) released in co-culture medium. C: Expression levels of apoptosis marker genes in HPCs. β-actin was used as a reference gene. * and **, *P*<0.05 and *P*<0.01 *vs* control value, respectively. †, *P*<0.05 *vs* eWAT co-culture group value.

In order to evaluate the possible different cytotoxic effect of iATEs and eATEs on HPCs, the number of detached HPCs and release of lactate dehydrogenase (LDH) into the media of HPC/ATE co-cultures were measured. As shown in Fig.5A, 24 h co-culturing of HPCs with iATEs, but not eATEs significantly increased the number of detached HPCs. Accordingly, the activity of LDH in the media from HPC/iATE co-cultures was significantly higher than that in the media collected from HPC/eATE co-cultures (Fig. 5B). The results were statistically significant after 24 h of co-culture (Fig 5A and B). Thus, iATEs appear to generate a more cytotoxic environment than eATEs.

To evaluate the effect of the ATEs on the attached HPCs, DNA and total mRNA of the attached HPCs were isolated for DNA fragmentation assay and real-time PCR analyses. In addition, fatty acid β-oxidation was measured in order to assay mitochondrial function. Mitochondrial fatty acid oxidation rate in the attached HPCs was not affected by co-culture, and DNA was not fragmented (data not shown), indicating that ATEs did not induce severe apoptosis during 24 h. However, co-culturing HPCs with iATEs led to a significantly increased expression of caspase-8 accompanied by reduced expression of the anti-apoptotic BCL-2 gene (Fig 5C).

### Insulin resistance properties of the HPCs co-cultured with ATEs

**Figure 6:**
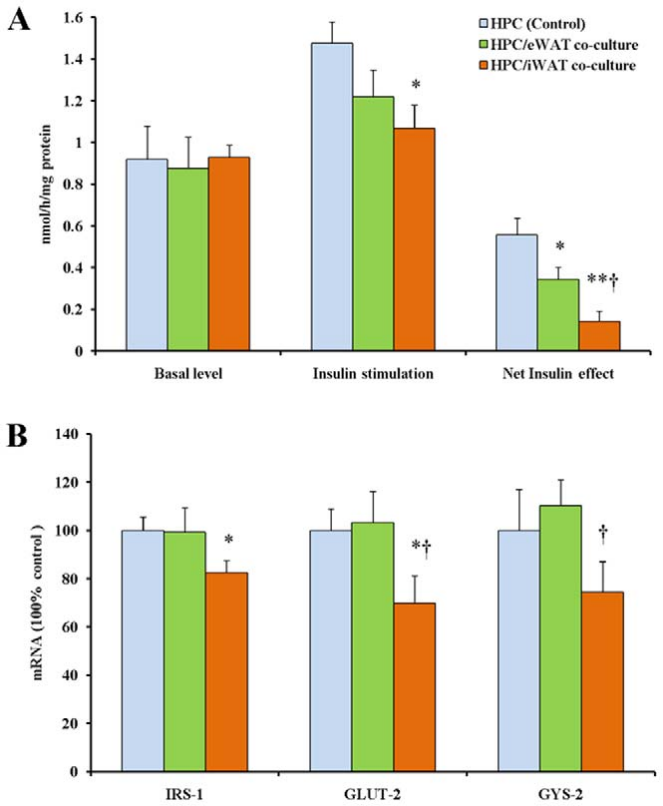
Insulin resistance properties of rat hepatocytes (HPC) co-cultured with adipose tissue explants from epididymal adipose tissue (eWAT) and inguinal adipose tissue (iWAT). A: Incorporation of [U-^14^C]glucose into glycogen in HPCs with the presence of insulin. B: Expression levels of genes regarding cellular glucose uptake and glycogen synthesis in HPCs. β-actin was used as a reference gene. * and **, *P*<0.05 and *P*<0.01 *vs* control value, respectively. †, *P*<0.05 *vs* eWAT co-culture group value.

Since insulin resistance is a major physiological consequence of obesity and also promoted by some inflammatory factors and adipokines [1], [2], [33], the insulin-stimulated incorporation of [^14^C]glucose into glycogen was determined in the attached HPCs cultured alone or co-cultured with iATEs and eATEs. As shown in Fig. 6A, glycogen synthesis was comparable between all groups at the basal level (no insulin stimulation). However, after insulin stimulation, the incorporation of glucose into glycogen in the HPCs was impaired when co-cultured with ATEs. To evaluate insulin resistance, the net insulin effect was calculated by subtracting basal glycogen synthesis levels from insulin-stimulated glycogen synthesis levels. Of note, insulin-stimulated glycogen synthesis and net insulin effect were significantly lower in HPCs co-cultured with iATEs than with eATEs. Reduced insulin-stimulated glycogen synthesis in the HPC co-cultured with iATEs, but not eATEs, was accompanied by reduced expression of insulin receptor substrate 1 (IRS-1), glucose transporter type 2 (GLUT-2) and glycogen synthase 2 (Gys-2) (Fig 6B). Together, these results indicate the factors released from iATEs induce higher insulin resistance than those from eATEs in the co-cultured HPCs.

## Discussion

The widespread epidemics of obesity and insulin resistance suggest that both conditions are closely linked. Dysfunctional adipose tissue, particularly as observed in obesity, is characterized by adipocyte hypertrophy, macrophage infiltration and increased secretion of cytokines. Thus, obesity has a strong inflammatory underpinning, and the degree of inflammation in adipose tissues appears to be central in regulating whole-body insulin-sensitivity. The increased levels of circulating cytokines produced by both adipocytes and infiltrating macrophages are causally linked to development of insulin resistance and diabetes [1], [2], [21]. Moreover, obese subjects have increased levels of plasma FFA that are known to cause insulin resistance in all major insulin target organs [4], [7]. The individual contributions of different adipose tissue depots to circulating cytokines and FFA are, however, still not fully elucidated.

In this study, by using a novel HPC/ATE co-culture model, we demonstrate that lipolysis, measured as glycerol release was similar in the ATEs isolated from inguinal and epididymal fat depots when cultured alone, but glycerol release was higher in the ATEs isolated from epididymal than from inguinal adipose tissue when co-cultured with hepatocytes. However, the ATEs from inguinal adipose tissue were far more efficient than the ATEs from epididymal adipose tissue in reducing insulin sensitivity in the co-cultured HPCs. In keeping with the finding that expression of inflammatory related genes and SVC marker genes was higher in iATE than eATE, and secretion of IL-6 and PGE_2_ was higher from inguinal ATEs than from epididymal ATEs, we suggest that the observed insulin-resistance in co-cultured HPCs are induced by cytokines rather than FFA. It is of note that there was no significant difference found in IL-6 when co-culturing iATEs or eATEs with HPCs. However, considering HPCs simultaneously have capacity to produce and degrade IL-6 [34], [35], the significant difference in IL-6 between iATEs and eATEs in individual culture would be alleviated by the inclusion of HPCs in co-culture. Given that adipocytes secrete low levels of cytokines compared to the adipose tissue SVCs [36], and macrophages are recognized as the main source of inflammatory cytokines such as TNF-α and IL-6 [21], we can not exclude that SVC, particularly macrophage, infiltration in iWAT contributes to the increased production of pro-inflammatory factors in iWAT. We also noticed that the possible residual blood cells, particularly neutrophils, would contribute to inflammatory factor release, however, taking into account that the expressions of neutrophil marker genes were comparable between iWAT and eWAT, the contribution of the resident neutrophils in ATEs to the inflammatory factor release is unlikely high. Indeed, in the present study, expressions of inflammatory markers and macrophage marker genes were significantly higher in SVCs isolated from iWAT than eWAT. Also, histological examination of iWAT and eWAT indicated a higher proportion of non-adipocytes in iWAT than eWAT. Thus, in studies using isolated adipocytes in co-culture, the cytotoxic effect of cytokines is potentially underestimated. By contrast, the cytotoxic effect of FFA may be overestimated as mature adipocytes are the main source of FFAs in circulation, whereas cytokines and eicosanoids are mainly produced by macrophages [20], [36], [37]. In the present study, co-culturing ATEs with HPCs indicated that the subcutaneous inguinal adipose tissue depot may elicit stronger responses on hepatocytes than visceral epididymal adipose tissue, which contrasts the general assumption that visceral fat confers more lipotoxic effects than subcutaneous fat [12], [13]. However, our results are in accordance with Villena et al [38] demonstrating a higher cytotoxic potential in inguinal than epididymal white adipose tissue (WAT) depots. Of note, they also reported that the differences were attributed to cells in the stromal-vascular fraction.

In a physiological situation, the location of adipose tissue may be of importance. However, our results support the notion that anatomically distinct fat depots are phenotypically distinct due to inherent cellular characteristics rather than because of differences in neural and/or blood supply to individual fat depots. It has been demonstrated that transplantation of subcutaneous adipose tissue to the visceral cavity improved insulin sensitivity in fasted mice suggesting that fat depots exhibit cell-autonomous effects in term of improved insulin action [13]. This result is consistent with the notion that expanding subcutaneous fat depots can protect against ectopic fat deposition and thereby prevent the adverse consequences of obesity on insulin sensitivity. However, it should be noted that these mice also exhibited decreased body weight and total fat mass. In an obese state, the location of adipose tissue may also in part determine the degree of adipose tissue inflammation and macrophage infiltration. Systemic effects of elevated circulating levels of pro-inflammatory cytokines, such as TNF-α and IL-6, are observed in obese individuals, whereas pro-inflammatory cytokines in lean subjects are believed to act in a paracrine manner [39]. Thus, we cannot exclude that eATEs from an obese rat would be more cytotoxic than iATEs. Also, the lipotoxic effect of FFA could be of more importance in the obese than in the lean state, as FFA release is dramatically elevated in obese compared to lean individuals (up to 300% increase) [40]. Potential under- and overestimation of the cytotoxic effect of cytokines and FFA by using isolated adipocytes in co-culture experiments, may account for the reported discrepancies in published results.

In conclusion, by using ATEs isolated from rats in different metabolic/physiological conditions in co-culture with primary hepatocytes, the co-culturing system described here could serve as a useful tool to examine adipose tissue-hepatocytes interaction in a setting resembling more closely different in vivo situations enabling analyses of depot-dependent effects on hepatocytes as a function of the nutritional and metabolic states of the adipose tissues.
